# Change in active travel and changes in recreational and total physical activity in adults: longitudinal findings from the iConnect study

**DOI:** 10.1186/1479-5868-10-28

**Published:** 2013-02-27

**Authors:** Shannon Sahlqvist, Anna Goodman, Ashley R Cooper, David Ogilvie

**Affiliations:** 1 Centre for Physical Activity and Nutrition Research (C-PAN) School of Exercise and Nutrition Sciences, Deakin University, Geelong, Australia; 2 Medical Research Council Epidemiology Unit & UKCRC Centre for Diet and Activity Research (CEDAR), Institute of Public Health, Cambridge, UK; 3 Faculty of Epidemiology and Population Health, London School of Hygiene and Tropical Medicine, London, UK; 4 Centre for Exercise, Nutrition and Health Sciences, School of Policy Studies, University of Bristol, Bristol, UK

**Keywords:** Active travel, Physical activity, Walking, Cycling, Longitudinal

## Abstract

**Background:**

To better understand the health benefits of promoting active travel, it is important to understand the relationship between a change in active travel and changes in recreational and total physical activity.

**Methods:**

These analyses, carried out in April 2012, use longitudinal data from 1628 adult respondents (mean age 54 years; 47% male) in the UK-based iConnect study. Travel and recreational physical activity were measured using detailed seven-day recall instruments. Adjusted linear regression models were fitted with change in active travel defined as ‘decreased’ (<−15 min/week), ‘maintained’ (±15 min/week) or ‘increased’ (>15 min/week) as the primary exposure variable and changes in (a) recreational and (b) total physical activity (min/week) as the primary outcome variables.

**Results:**

Active travel increased in 32% (n=529), was maintained in 33% (n=534) and decreased in 35% (n=565) of respondents. Recreational physical activity decreased in all groups but this decrease was not greater in those whose active travel increased. Conversely, changes in active travel were associated with commensurate changes in total physical activity. Compared with those whose active travel remained unchanged, total physical activity decreased by 176.9 min/week in those whose active travel had decreased (adjusted regression coefficient −154.9, 95% CI −195.3 to −114.5) and was 112.2 min/week greater among those whose active travel had increased (adjusted regression coefficient 135.1, 95% CI 94.3 to 175.9).

**Conclusion:**

An increase in active travel was associated with a commensurate increase in total physical activity and not a decrease in recreational physical activity.

## Background

A growing body of evidence for the associations between active travel and reduced risk of cardiovascular mortality, overweight and obesity, hypertension and type 2 diabetes, supports its promotion as a way of improving public health [1]. These health benefits are likely due, in large part, to the extent to which active travel results in greater total physical activity.

The findings of a recent review [2] and of other studies [3-7] suggest, fairly convincingly, that in adults active travel is associated with greater self-reported total physical activity [8-10] accelerometer-determined total physical activity [3,5,11] and total step counts [4,7]. Furthermore, studies that have examined associations between active travel and recreational physical activity in particular suggest that active travel is not necessarily undertaken as a substitute for recreational physical activity [12,13]. In some studies active travel was associated with greater time spent in recreational physical activity [11,14-16]. For example, adults in one study who travelled mainly on foot or by bike reported a statistically significant additional 15 min/day in recreational physical activity compared with adults who never or rarely travelled by bike or on foot [14].

Although encouraging, previous research has been limited by the use of crude measures of active travel (i.e. usual travel mode) and the investigation of only one travel purpose or mode (i.e. commuting). A UK study recently extended this work by simultaneously collecting more detailed information on travel and recreational physical activity. Findings indicated that active travel was done in addition to, rather than instead of, recreational physical activity and suggested a dose–response relationship between active travel and total physical activity [17].

Nevertheless, there is currently little evidence that an *increase* in active travel results in a commensurate increase in total physical activity. It is possible that an increase in active travel might be compensated for by a decrease in activity in other domains. For example, a person may start walking to work and subsequently forego their morning recreational walk. Alternatively, as has been suggested in studies with children, an increase in active travel may result in greater total physical activity, either because recreational physical activity remains unchanged [18] or because active travel encourages physical activity in other domains [19].

In adults, it is important to establish the association between a change in active travel and change in total physical activity to strengthen our understanding of the population health impact of *promoting* active travel. This paper therefore builds on previous cross-sectional research in the UK population-based iConnect study [17] by examining the longitudinal association between a change in active travel and changes in recreational and total physical activity.

## Methods

### Study design & participant recruitment

The present analyses use data collected from the iConnect study. Full details of the study methods, including the survey instrument and its development, are provided elsewhere [20,21]. In brief, the iConnect study is a longitudinal study investigating the impact of newly constructed infrastructure for walking and cycling on travel, physical activity and carbon emissions. In April 2010, adults aged over 18 years (n=22500) residing in the three core study areas (Cardiff, Kenilworth and Southampton) were randomly selected from the edited electoral register and sent a survey which asked about their travel and physical activity behaviour and included standard sociodemographic questions. One year later (April 2011), the respondents were sent a second copy of the survey. Ethical approval was granted by the University of Southampton Ethics Committee.

### Exposure measures

At both time points, respondents were asked to recall all journeys made over the previous seven days for journeys to and from work, to and from a place of study (categorised as commuting travel), for shopping and personal business and for visiting friends or relatives or other social activities (categorised as non-commuting travel). For each of these purposes, respondents recalled the number of journeys made as well as the total time (min/week) spent and distance (miles/week) travelled using each of six modes of transport (walking, cycling, bus, train, car and ‘other’). Participants were instructed to assign each return journey to a single ‘main purpose’ and report all modes used on each journey. Where a respondent had reported the distance but not the time travelled, time was imputed using the sample mean of the observed speed for each mode/purpose combination. Time spent walking and cycling for commuting and non-commuting purposes, and for all purposes, was summed to provide aggregate measures of active travel.

Change in active travel was calculated by subtracting weekly time spent in active travel at baseline from weekly time spent in active travel at follow-up. To account for the likely imprecision in the measure respondents with a change value of ±15 min/week were categorised as having maintained their active travel, those with a value of >15 min/week as having increased their active travel, and those with a value of <−15 min/week as having decreased their active travel. To explore possible dose–response relationships, respondents were also categorised according to the magnitude of this change using cut points of ±15, ±45, ±90 and ±135 min/week. Change values were also computed for four separate sub-exposures: change in (a) commuting active travel, (b) non-commuting active travel, (c) walking for all transport purposes and (d) cycling for all transport purposes.

### Outcome measures

Recreational physical activity was assessed by asking participants to recall the number of sessions and total time spent over the past seven days in four types of recreational activities: walking, cycling, other moderate-intensity physical activity and other vigorous-intensity physical activity. The questions were adapted from the short form of the International Physical Activity Questionnaire (IPAQ) so that they specifically asked about recreational walking and cycling separately [22]. Respondents were asked to recall the number of sessions and total time spent over the past seven days in the four types of recreational activities. Data cleaning procedures similar to those applied to the short IPAQ were used whereby for each activity category, data were truncated at 1260 min (21 h/week) and respondents who reported greater than 6720 min (16 h/day) were excluded [22]. Total recreational physical activity (min/week) was computed by summing time spent in these four activities. Total physical activity (min/week) was computed by summing time spent in all active travel and in recreational physical activity.

### Sociodemographic and other characteristics

Respondents were categorised according to their sex and baseline age, body mass index (BMI, computed from self-reported height and weight), highest educational qualification, employment status, ethnicity, housing tenure, household car access and the presence of children in the household.

### Analyses

Analyses were carried out in April 2012 using STATA/SE 11.0. Linear regression models were first fitted with change in (a) recreational and (b) total physical activity as the outcome variables and with change in active travel (decreased, maintained, increased) as the exposure variable. Similar models were then fitted with change in active travel entered as a continuous variable (min/week). All models were adjusted for individual (age, sex, ethnicity, education, employment status, BMI) and household (housing tenure, income, car ownership, presence of children in household) level characteristics. Age and BMI were entered as linear terms and the remainder entered using the categories shown in Table 1. The models reported here were not adjusted for baseline levels of physical activity on the grounds that doing so can introduce bias if exposures and outcomes are measured with error or are prone to short-term fluctuations, or if average baseline values of the outcome measure differ between exposure categories [23,24]. It appeared likely that some or all of these conditions could apply to these analyses. By way of sensitivity analyses, all models were repeated with adjustment for baseline physical activity; this made no substantive difference to the findings (results not shown).

**Table 1:** **Sociodemographic characteristics of respondents by change in active travel**^**a**^

**Characteristic**	**All n=1628**		**Active travel decreased n=565**		**Active travel was maintained n=534**		**Active travel increased n=529**	
	**mean (SD)**		**mean (SD)**		**mean (SD)**		**mean (SD)**	
**Age (years)**	54.4 (16.3)		52.2 (16.6)		59.2 (14.8)		52.9 (16.3)	
**BMI (kg/m**^**2**^**)**	25.7 (4.6)		25.3 (4.5)		26.5 (4.7)		25.5 (4.6)	
	**n**	**%**	**n**	**%**	**n**	**%**	**n**	**%**
**Site**								
Cardiff	521	32.0	190	33.6	160	30.0	171	32.3
Kenilworth	651	40.0	193	34.2	113	48.9	197	37.2
Southampton	456	28.0	182	32.2	113	21.2	161	30.4
**Sex**								
Male	757	46.5	252	44.6	259	48.5	246	46.5
Female	871	53.5	313	55.4	275	51.5	283	53.5
**Ethnicity**								
White	1560	96.4	537	95.6	513	96.4	510	97.1
Other	59	3.6	25	4.5	19	3.6	15	2.9
**Education**								
University qualification	672	41.5	2556	45.5	188	35.5	229	43.3
‘A’ Level	261	16.1	93	16.6	85	16.0	83	15.7
GCSE	300	18.5	92	16.4	109	20.6	99	18.7
No formal qualification	386	23.8	120	21.4	148	27.9	118	22.3
**Housing Tenure**								
Owned	1374	84.4	464	82.2	482	90.3	428	80.9
Rented (private landlord)	135	8.3	56	9.9	23	4.3	56	10.6
Rented (local authority)	88	5.4	31	5.5	21	3.9	36	6.8
Other	31	1.9	14	2.5	8	1.5	9	1.7
**Employment**								
Full-time	641	39.4	208	36.8	219	41.0	214	40.5
Part-time	251	15.4	90	15.9	84	15.7	77	14.6
Student	49	3.0	25	4.4	9	1.7	15	2.8
Retired	560	34.6	193	34.2	197	36.9	174	32.9
Other	123	7.6	49	8.7	25	4.7	49	9.3
**Household income**								
>£40,000	559	37.0	186	35.7	195	38.9	178	36.4
£20,001 to £40,000	501	33.2	178	34.2	159	31.7	164	33.5
≤£20,000	451	29.9	157	30.1	147	29.3	147	20.6
**No. of cars per adult**								
None	168	10.3	68	12.1	33	6.2	67	12.7
<1	630	38.8	228	40.5	200	37.5	202	38.2
≥1	828	50.9	267	47.4	301	56.4	260	49.2
**Children <16 years**								
No	1345	82.6	459	81.2	456	85.4	430	81.3
Yes	283	17.4	106	18.8	78	14.6	99	18.7

^a^Numbers do not always sum to totals due to missing responses.

BMI: Body mass index.

The number of cases with missing data for sociodemographic covariates at baseline ranged from 16 (for sex) to 260 (for household income). Where possible, these missing baseline data were replaced with follow-up measures of the same variables for between 1 and 106 cases (depending on the variable), which reduced the number of cases with missing data for a given variable to between 0 and 114. Multiple imputation using chained equations (with five imputations) under the assumption of missing at random was used to impute the remaining missing data. All covariates and outcome variables entered into the regression models were included in the imputation model. Substantive findings were unchanged in sensitivity analyses using complete case analysis and/or excluding income, the variable with the highest level of missing data.

## Results

### Descriptive statistics

A completed baseline survey was returned by 3516 (16%) respondents, of whom 1885 (54%) returned a completed follow-up survey one year later. Of these, 232 had missing physical activity or travel data and 25 had extreme differences (>600 min/week) in active travel between baseline and follow-up. Excluding these cases left 1628 respondents. The likelihood of being included in follow-up analysis was associated with being older (56.1% of baseline respondents aged over 65 were included, compared with 30.1% of 18-34-year-olds), owning a home (52.7%, versus 26.3% of those who rented privately) and having access to a car (48.4%, versus 31.5% of those with no car; see Additional file 1). Comparisons with local and national data suggested that on average, respondents were older and more likely to have completed higher education, to own their home, to have access to a car, to be of a normal weight and to be economically active, and less likely to have children living at home, than the general population (see Additional file 2).

Respondents had a mean age of 54 years and just over half were female (Table 1). Total time spent in active travel changed little over one year, with a mean change of −4.4 min/week (95% CI −11.7 to 2.8). Recreational physical activity declined by a mean of 28.2 min/week (95% CI −43.4 to −12.9) resulting in a mean decrease in total physical activity of −32.6 min/week (95% CI −49.8 to −15.4). At the individual level, weekly active travel increased in 32% (n=529), was maintained in 33% (n=534) and decreased in 35% (n=565) of respondents over the year.

Respondents whose active travel decreased reported an average of 230.8 min/week (95% CI 214.5 to 247.2) of active travel at baseline which decreased to 93.6 min/week (95% CI 82.2 to 105.0) at follow-up. Conversely, respondents categorised as increasing their active travel reported an average of 85.3 min/week (95% CI 75.7 to 95.0) at baseline, increasing to 218.6 min/week (95% CI 204.0 to 233.2) at follow-up. Those who maintained their active travel reported an average of 31.5 min/week (95% CI 24.3 to 30.7) of active travel at baseline. Of the ‘maintainers’ the majority (n=382) reported no active travel, while the remaining (n=152) reported an average of 110.7 min/week (95% CI 90.3 to 131.1).

### Association between change in active travel and change in recreational and total physical activity

**Figure 1:**
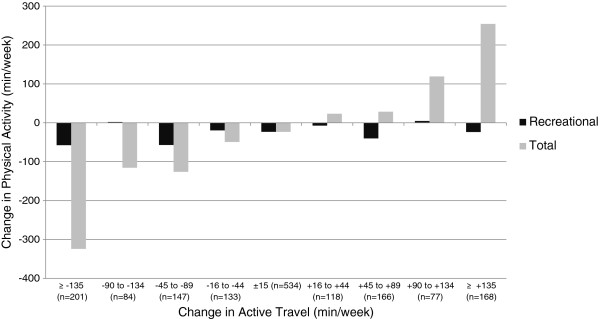
Association between subcategories of change in active travel and change in (a) recreational and (b) total physical activity.

Table 2 summarises descriptive statistics and linear regression models showing the association between change in active travel and change in recreational and total physical activity. The comparison of unadjusted mean changes is further described in Figure 1, which shows the relationship between the magnitude of change in active travel and the magnitude of change in total physical activity.

**Table 2:** Changes in active travel and (a) recreational and (b) total physical activity (min/week)

	**n**	**Recreational PA**				**Total PA**			
		**T1 mean (95% CI)**	**T2 mean (95% CI)**	**Mean change (95% CI)**	**Regression coefficient**^**a **^**(95% CI)**	**T1 mean (95% CI)**	**T2 mean (95% CI)**	**Mean change (95% CI)**	**Regression coefficient**^**a **^**(95% CI)**
Active travel decreased	565	319.7 (293.2, 346.2)	280.0 (254.6, 305.4)	−39.7 (−64.6, -14.8)	−18.2 (−56.2, 19.9)	550.6 (517.0, 584.2)	373.7 (344.2, 403.2)	−176.9 (204.3, -149.5)	−154.9 (−195.3, -144.5)
Active travel maintained	534	275.3 (247.1, 303.6)	252.3 (224.5, 280.1)	−23.1 (−49.4, 3.2)	0	307.2 (277.7, 336.8)	283.8 (254.5, 313.0)	−23.5 (−49.8, 2.9)	0
Active travel increased	529	320.9(288.4, 353.5)	299.9 (273.5, 326.2)	−21.0 (−49.1, 7.0)	1.8 (−36.6, 40.2)	406.2 (371.3, 441.2)	518.5 (486.3, 550.7)	112.2 (81.9, 142.6)	135.1 (94.3, 175.9)

^**a**^Linear regression adjusted for age, sex, ethnicity, education, employment, BMI, household income, housing tenure, household car access and children living at home. In all models maintenance of active travel was set as the reference category. 95%CI: 95% Confidence Intervals; T1 mean: mean at baseline; T2 mean: mean at follow-up; PA: Physical Activity.

Small decreases in recreational activity were seen across all groups (Figure 1), and there was no evidence that this varied by category of change in active travel (*p* = 0.47 for heterogeneity; Table 2). Furthermore, there was no correlation (r=0.05) between change in active travel and change in recreational physical activity. In fact, the direction of the non-significant effect was the opposite to that which would be expected if activity compensation were operating, with a larger decrease observed in those whose active travel decreased (mean decrease 39.7 min/week) than in those whose active travel increased (mean decrease 21.0 min/week).

By contrast, there was strong evidence that a change in active travel was associated with a change in total physical activity. Those whose active travel increased had a significant increase in their total physical activity compared with maintainers (adjusted regression coefficient 135.1, 95% CI 94.3 to 175.9), while those whose active travel decreased had a corresponding decrease in total physical activity compared with maintainers (adjusted regression coefficient −154.9, 95% CI −195.3 to −114.5; Table 2). This association showed an approximately dose–response relationship across the full range of change in active travel categories (Figure 1). Similar results were also seen when active travel was entered as a continuous variable.

By way of sensitivity analyses we explored whether the associations differed according to respondents’ absolute levels of active travel at baseline and follow-up, categorising respondents as: (a) maintaining zero active travel, (b) maintaining some active travel, (c) reducing their active travel but continuing to report some, (d) reducing their active travel to zero, (e) taking up active travel, or (f) increasing their active travel from non-zero at baseline. In linear regression models, the associations remained consistent with the main analyses. That is, a change in active travel was associated with a change in total physical activity regardless of whether the change was from ‘zero’ or from ‘some’ active travel at baseline (see Additional file 3).

Tables 3 and 4 report the results from linear regression models examining changes in active travel by purpose (commuting vs. non-commuting) and by mode (walking vs. cycling). Associations remained largely unchanged when examining changes in commuting and non-commuting active travel separately (Table 3). In separate analysis restricted to respondents who reported commuting at both time points, an increase in active travel was associated with a significant increase in total physical activity (regression coefficient 125.0, 95% CI 66.2 to 183.9) whereas a decrease in active travel was associated with a significant decrease (regression coefficient −148.2, 95% CI −206.8 to −89.7). The associations also held when examining changes in utility walking and cycling separately (Table 4). The observation that an increase in walking was not associated with a corresponding decrease in cycling and vice versa suggests that, in general, respondents were not switching between active modes of transport.

**Table 3:** Changes in purpose-specific active travel and (a) recreational and (b) total physical activity (min/week)

**Commuting active travel**													
		**Non-Commuting travel PA**				**Recreational PA**				**Total PA**			
	**N**	**T1 mean (95% CI)**	**T2 mean (95% CI)**	**Mean change (95% CI)**	**Regression coefficient**^**a **^**(95 % CI)**	**T1 mean (95% CI)**	**T2 mean (95% CI)**	**Mean change (95% CI)**	**Regression coefficient**^**a **^**(95% CI)**	**T1 mean (95% CI)**	**T2 mean (95% CI)**	**Mean change (95% CI)**	**Regression coefficient**^**a **^**(95% CI)**
Active travel decreased	254	87.1 (73.4, 100.9)	104.3 (83.5, 125.1)	17.2 (−2.8, 37.2)	26.9 (7.9, 45.8)	316.1 (272.1, 360.1)	266.8 (232.8, 300.8)	−49.3 (−87.9, -10.8)	−37.2 (−82.4, 8.0)	559.0 (513.5, 624.4)	424.7 (378.4, 470.9)	−144.3 (−189.6, -98.9)	−120.8 (−171.1, -70.4)
Active travel maintained	1154	76.3 (69.1, 83.6)	70.2 (63.4, 77.0)	−6.1 (−13.3,1.1)	0	303.3 (283.3, 323.2)	281.3 (262.2, 300.5)	−21.9 (−40.3, -3.5)	0	387.5 (365.2, 409.9)	338.2 (338.1, 381.1)	−27.9 (−48.0, -7.8)	0
Active travel increased	220	74.9 (60.8, 88.9)	69.0 (57.0, 81.0)	−5.9 (−20.9, 9.2)	3.1 (−16.9, 23.2)	305.4 (260.6, 350.2)	268.7 (233.3, 304.2)	−36.6 (−74.4, 1.1)	−25.7 (−73.5, 22.2)	446.8 (395.7, 498.0)	518. (473.3, 563.6)	71.6 (28.7, 114.6)	93.2 (40.0, 146.5)
**Non-Commuting active travel**													
		**Commuting PA**				**Recreational PA**				**Total PA**			
	**N**	**T1 mean (95% CI)**	**T2 mean (95% CI)**	**Mean change (95% CI)**	**Regression coefficient**^**a **^**(95% CI)**	**T1 mean (95% CI)**	**T2 mean (95% CI)**	**Mean change (95% CI)**	**Regression coefficient**^**a **^**(95% CI)**	**T1 mean (95% CI)**	**T2 mean (95% CI)**	**Mean change (95% CI)**	**Regression coefficient**^**a **^**(95% CI)**
Active travel decreased	502	53.3 (44.1, 62.4)	49.0 (39.6, 58.4)	−4.3 (−11.9, 3.3)	−10.0 (−20.6, 0.6)	329.3 (300.2, 358.3)	288.9 (260.9, 316.9)	−40.4 (−67.1, -13.6)	−24.7 (−62.0, 12.7)	557.1 (522.1, 592.1)	390.1 (358.3, 422.0)	−167.0 (−196.7, -137.3)	−157.9 (−198.5, -117.4)
Active travel maintained	637	23.1 (18.0, 28.2)	29.6 (23.0, 36.1)	6.5 (1.0, 12.0)	0	268.5 (243.6, 293.3)	253.2 (228.8, 277.7)	−15.2 (−38.1, 7.6)	0	308.6 (282.5, 334.8)	299.8 (273.3, 326.2)	−8.9 (−32.7, 15.0)	0
Active travel increased	489	50.0 (39.0, 61.0))	39.3 (31.6, 47.0)	−10.7 (−20.3, -1.1)	−16.4 (−27.0, -5.8)	329.5 (295.1, 363.9)	297.0 (269.5, 324.5)	−32.5 (−63.1, -2.0)	−15.5 (−52.9, 22.0)	437.2 (398.3, 476,1)	511.5 (477.2, 545.9)	74.4 (40.6, 108.1)	85.5 (44.9, 126.1)

^**a**^Linear regression adjusted for age, sex, ethnicity, education, employment, BMI, household income, housing tenure, household car access and children living at home. 95%CI: 95% Confidence Intervals; PA: Physical activity.

**Table 4:** Changes in mode-specific active travel and (a) recreational and (b) total physical activity (min/week)

**Walking for transport**													
		**Cycling for transport**				**Recreational PA**				**Total PA**			
	**n**	**T1 mean (95% CI)**	**T2 mean (95% CI)**	**Mean change (95% CI)e**	**Regression coefficient**^**a **^**(95 % CI)**	**T1 mean (95% CI)**	**T2 mean (95% CI)**	**Mean change (95% CI)**	**Regression coefficient**^**a **^**(95% CI)**	**T1 mean (95% CI)**	**T2 mean (95% CI)**	**Mean change (95% CI)**	**Regression coefficient**^**a **^**(95% CI)**
Walking decreased	554	19.2 (13.9, 24.6)	719.2 (13.7, 24.6)	−0.1 (−4.1, 4.0)	0.75 (−5.4, 6.9)	317.3 (290.9, 343.6)	283.0 (257.7, 308.3)	−34.2 (−58.6, -9.9)	−11.5 (−49.2, 26.3)	545.2 (412.0, 578.4)	380.5 (350.5, 410.5)	−164.7 (−191.6, -137.8)	−141.5 (−182.0, -101.0)
Walking maintained	564	9.1 (5.5, 12.8)	9.2 (5.9, 12.5)	0.1 (−2.9, 3.1)	0	279.8 (251.8, 307.9)	255.5 (228.3, 282.7)	−24.3 (−50.1, 1.4)	0	315.0 (285.7, 344.3)	290.6 (262.2, 319.0)	−24. 4 (−50.4, 1.6)	0
Walking increased	510	16.6 (10.7, 22.5)	18.7 (12.7, 24.7)	2.1 (−3.5, 7.7)	3.2 (−3.0, 9.5)	321.3 (288.1, 354.4)	295.4 (268.4, 322.4)	−25.8 (−55.2, 3.5)	−0.7 (−39.0, 37.7)	412.4 (375.9, 448.9)	514.2 (481.0, 547.4)	101.8 (69.3, 134.3)	127.0 (85.8, 168.2)
**Cycling for Transport**													
		**Walking for Transport**				**Recreational PA**				**Total PA**			
	**n**	**T1 mean (95% CI)**	**T2 mean (95% CI)**	**Mean change (95% CI)**	**Regression coefficient**^**a **^**(95% CI)**	**T1 mean (95% CI)**	**T2 mean (95% CI)**	**Mean change (95% CI)**	**Regression coefficient**^**a **^**(95% CI)**	**T1 mean (95% CI)**	**T2 mean (95% CI)**	**Mean change (95% CI)**	**Regression coefficient**^**a **^**(95% CI)**
Cycling decreased	96	139.2 (107.9, 170.5)	127.9 (96.7, 159.1)	−11.3 (−40.7, 18.0)	−5.6 (−35.3, 24.1)	492.9 (403.1, 582.8)	390.6 (316.5, 464.7)	−102.3 (−185.0, -19.7)	−79.4 (−145. 2, -13.7)	797.9 (688.6, 907.3)	581.6 (489.8, 673.4)	−216.3 (−311.2, -121.5)	−187.6 (−261.1, -114.1)
Cycling maintained	1418	99.3 (91.6, 106.9)	94.8 (87.5, 102.1)	−4.5 (−11.8, 2.8)	0	287.3 (270.2, 304.4)	261.9 (245.8, 278.0)	−25.4 (−41.1, -9.7)	0	388.3 (369.1, 407.4)	358.4 (340.0, 376.7)	−29.9 (−47.4, -12.4)	0
Cycling increased	114	124.6 (86.4, 162.7)	117.0 (85.1, 149.0)	−7.5 (−32.5, 17.5)	1.7 (−26.0, 29.4)	374.4 (299.0, 449.8)	374.2 (319.0, 429.5)	−0.2 (−64.9, 64.6)	15.4 (−45.8,.76.7)	551.4 (462.0, 640.8)	639.6 (566.5, 712.8)	88.3 (17.5, 159.0)	113.3 (44.9, 181.7)

^**a**^Linear regression adjusted for age, sex, ethnicity, education, employment, BMI, household income, housing tenure, household car access and children living at home 95%CI: 95% Confidence Intervals; PA: Physical activity.

## Discussion

Findings from this longitudinal study of UK adults demonstrate that a change in active travel is associated with a commensurate change in total physical activity. The pattern of associations was consistent with a dose–response relationship. These findings build on those of cross-sectional studies which have reported positive associations between total physical activity and use of active travel in general [10] and public transport [4-6] and utility cycling in particular [3,9]. They are also consistent with those of recent longitudinal studies which reported increases in objectively-measured total physical activity in children who changed from a motorised to active mode of travel to school [18,25].

While recreational physical activity decreased in all groups over the year, there was no evidence that this decrease was greater in those whose active travel had increased. Previous studies have shown that adults do not appear to participate in active travel to account for their lack of recreational physical activity [12,13]. Moreover there is some evidence to suggest that adults who walk or cycle as a means of transport may actually participate in more recreational physical activity [14]. This study has further demonstrated that an increase in active travel does not appear to compensate for a decrease in recreational physical activity. This important observation suggests that if adults can be encouraged to participate in active travel they are likely to do so in addition to their other physical activity, thereby increasing their overall physical activity.

By including a detailed measure of travel behaviour, this study also found that the association between active travel and physical activity held when examining changes in commuting and non-commuting active travel as well as when examining changes in the distinct behaviours of walking and cycling for transport. Walking and cycling may be more or less attractive or feasible for different people in different circumstances. Our findings suggest that in principle, strategies to promote walking, cycling or both, whether for commuting or for other purposes, may all hold promise as a means of increasing physical activity overall.

### Strengths and limitations

To the best of our knowledge this is the first study to examine the association between a change in active travel and change in total physical activity in adults. It builds further on previous work by examining the association in a relatively large sample and by including detailed disaggregated measures of both travel and recreational physical activity, although these were self-reported and subject to possible recall bias. To capture the necessary level of detail two existing instruments were adapted: travel behaviour was assessed using a seven-day recall instrument and recreational physical activity was assessed using a purposively adapted version of the IPAQ. Although the IPAQ has been extensively used, like many physical activity questionnaires its criterion validity with respect to objectively measured physical activity is modest and it tends to overestimate physical activity [26]. It is likely that the measurement error associated with the IPAQ and the seven-day travel instrument attenuated effect sizes. It is hard to know the relative magnitude of this potential bias, although it seems unlikely that it would be sufficient to disguise a genuine, substantial decrease in recreational physical activity among those whose active travel increased. Moreover, the criterion validity of the two measures when used together remains to be determined. Consequently, the extent to which respondents may have reported their walking and cycling as both recreation and transport remains unknown. Notably, however, change in active travel was not correlated with change in recreational physical activity, suggesting that ‘double counting’ is unlikely to have had much effect in this study. In addition, the measure of total physical activity derived for these analyses did not include time spent in occupational or household physical activity. It seems less plausible that activity compensation would occur in these domains than with respect to recreational physical activity given that in general, people have more control over the latter than the former. Finally, the items used to measure travel behaviour did not ask about the perceived intensity of the walking and cycling. This may be important in light of evidence suggesting greater health benefits from cycling at vigorous perceived intensity than from cycling at light or moderate perceived intensity [27].

The study achieved an acceptable follow-up rate of 54%. Nonetheless, the original response to the survey was low, albeit comparable to that achieved in other surveys implemented for the purpose of evaluating natural experiments [28,29]. Although the travel patterns of respondents were comparable to those of adults participating in the recent UK National Travel Survey (NTS) [17], the sample was composed of respondents who were older and more likely to have completed higher education than the local populations from which they were drawn. Baseline data also suggest that respondents were more likely to meet the current physical activity recommendations than respondents in the 2008 Health Survey for England (HSE) [30], although these differences may reflect the use of a much more detailed, disaggregated measure of travel and recreational physical activity than those used in the HSE. Given the differences between the sample and the general population, the observed associations may not be generalizable to the wider population.

Finally, while the findings indicate that a change in active travel is associated with a change in overall physical activity, the direction of causality is unknown. It is not possible to conclude from these results whether an increase in respondents’ overall physical activity was reflected in an increase in walking or cycling, or whether an increase in walking or cycling was followed by an increase in overall physical activity.

## Conclusion

This study suggests that an increase in active travel is associated with a commensurate increase in total physical activity in adults. Future research using more detailed, objective measures of both active travel and recreational physical activity are needed to confirm and extend these findings. If they were to be replicated, they would suggest that promoting active travel has considerable public health potential as a strategy for increasing overall levels of physical activity in the adult population.

## Competing interests

The authors declare that they have no competing interests.

## Authors’ contributions

SS led the analysis and the writing of the manuscript. AG advised on analysis. SS, AC & DO contributed to the design of the study. All authors contributed to the interpretation of the findings and critical revision of the manuscript and approved the final version.

## Supplementary Material

Additional file 1 **Predictors of being included in analyses (N=1628) based on response to baseline survey (N=3516).** A table comparing the sociodemographic characteristics of respondents to the baseline survey (n=3516) to those included in these analyses (n=1628). Click here for file

Additional file 2 **Comparison of study population (N=1628) with the general population.** A table comparing the respondents included in the analyses with the general population on key sociodemographic characteristics. Click here for file

Additional file 3 **Association between subcategories of change in active travel and change in (a) recreational and (b) total physical activity.** The findings from supplementary analyses of the association between subcategories of change in active travel and (a) recreational and (b) total physical activity. Click here for file
