# Supplementary material for: Change in active travel and changes in recreational and total physical activity in adults: longitudinal findings from the iConnect study
Source: Int J Behav Nutr Phys Act. 2013 Feb 27;10:28. doi: 10.1186/1479-5868-10-28 (PMC3598920; doi:10.1186/1479-5868-10-28)
Supplement: Additional file 1 — Predictors of being included in analyses (N=1628) based on response to baseline survey (N=3516). A table comparing the sociodemographic characteristics of respondents to the baseline survey (n=3516) to those included in these analyses (n=1628). [file 1479-5868-10-28-S1.doc]

**Additional file 1**

**Predictors of being included in analyses (N=1628) based on response to baseline survey** (N=3516)

| **Variable** | **Level** | **N at** | **% included** | **RR (95%CI) for inclusion at follow-up** | |
| --- | --- | --- | --- | --- | --- |
|  |  | **baseline** | **in follow-up** | **Unadjusted** | **Adjusted**† |
| Site | Southampton | 1,127 | 40.5 | 1*** | 1 |
|  | Cardiff | 1,124 | 46.4 | 0.79 (0.67, 0.93) | 1.01 (0.84, 1.22) |
|  | Kenilworth | 1,265 | 51.5 | 1.23 (1.04, 1.44) | 1.06 (0.89, 1.26) |
| Sex | Female | 1,924 | 48.2 | 1 | 1 |
|  | Male | 1,570 | 45.3 | 0.88 (0.78, 1.02) | 0.96 (0.82, 1.11) |
| Age (years) | 18-34 | 793 | 30.1 | 1*** | 1*** |
|  | 35-49 | 803 | 44.7 | 1.87 (1.52, 2.30) | 1.53 (1.20, 1.94) |
|  | 50-64 | 1,003 | 54.4 | 2.77 (2.27, 3.37) | 1.94 (1.52, 2.47) |
|  | >65 | 856 | 56.1 | 2.96 (2.42, 3.63) | 1.94 (1.40, 2.69) |
| Ethnicity | White | 3,268 | 47.7 | 1*** | 1 |
|  | Non-White | 185 | 31.9 | 0.51 (0.37, 0.70) | 0.73 (0.52, 1.02) |
| Any child | No | 2,771 | 48.5 | 1*** | 1** |
| under 16 | Yes | 708 | 40.0 | 0.71 (0.60, 0.83) | 0.75 (0.61, 0.93) |
| Education | Degree | 1,377 | 48.8 | 1 | 1 |
|  | A-level | 601 | 43.4 | 0.81 (0.66, 0.98) | 0.92 (0.74, 1.13) |
|  | GCSE | 647 | 46.4 | 0.91 (0.75, 1.09) | 0.82 (0.67, 1.00) |
|  | No formal | 799 | 48.3 | 0.98 (0.86, 1.06) | 0.85 (0.68, 1.05) |
| Annual | >£40,000 | 1,084 | 51.6 | 1 | 1 |
| household | £20,001-40,000 | 981 | 51.1 | 1.18 (0.99, 1.41) | 0.93 (0.76, 1.12) |
| Income | ≤£20,000 | 962 | 46.9 | 1.21 (1.01, 1.44) | 0.82 (0.65, 1.03) |
| Employment | Full-time | 1,406 | 45.6 | 1*** | 1** |
| Status | Part-time | 481 | 52.2 | 1.30 (1.06, 1.60) | 1.1 (0.89, 1.39) |
|  | Student | 223 | 22.0 | 0.34 (0.24, 0.47) | 0.62 (0.42, 0.90) |
|  | Retired | 991 | 56.9 | 1.58 (1.34, 1.86) | 1.13 (0.87, 1.47) |
|  | Other | 369 | 33.3 | 0.60 (0.47, 0.76) | 0.74 (0.56, 0.96) |
| Housing tenure | Owned  Rent privately  Rent from local authority  Other | 2606  513  269  78 | 52.7  26.3  32.7  39.7 | 1***  0.32 (0.26, 0.40)  0.44 (0.33, 0.57)  0.59 (0.37, 0.94) | 1***  0.54 (0.42, 0.69)  0.59 (0.43, 0.79)  0.70 (0.43, 1.13) |
| Cars per adult | No cars | 534 | 31.5 | 1*** | 1** |
| in household | <1 car per adult | 1,302 | 48.4 | 2.04 (1.65, 2.52) | 1.58 (1.24, 2.01) |
|  | ≥1 cars per adult | 1,651 | 50.2 | 2.19 (1.78, 2.69) | 1.48 (1.16, 1.88) |
| Weight status | Normal | 1,694 | 46.6 | 1 | 1 |
|  | Overweight | 1,151 | 49.2 | 1.11 (0.95, 1.29) | 0.93 (0.79, 1.09) |
|  | Obese | 477 | 44.9 | 0.93 (0.76, 1.14) | 0.83 (0.66, 1.03) |
| Walking or | None | 550 | 47.5 | 1 | 1 |
| cycling for | 1-149 | 920 | 48.0 | 1.02 (0.83, 1.27) | 1.24 (0.99, 1.55) |
| transport or | 150-299 | 753 | 49.8 | 1.10 (0.88, 1.39) | 1.33 (1.05, 1.70) |
| recreation in past | 300-449 | 489 | 48.3 | 1.02 (0.91, 1.32) | 1.32 (1.01, 1.72) |
| week (min) | ≥450 | 698 | 45.0 | 0.91 (0.72, 1.13) | 1.10 (0.86, 1.40) |

*p<0.05, **p<0.01, ***p<0.001 for heterogeneity. RR=rate ratio, CI=confidence interval.

† Adjusted for age, ethnicity, housing tenure and employment status. Selected as the measures most strongly associated with retention in univariable analyses.
