# Supplementary material for: Change in active travel and changes in recreational and total physical activity in adults: longitudinal findings from the iConnect study
Source: Int J Behav Nutr Phys Act. 2013 Feb 27;10:28. doi: 10.1186/1479-5868-10-28 (PMC3598920; doi:10.1186/1479-5868-10-28)
Supplement: Additional file 2 — Comparison of study population (N=1628) with the general population. A table comparing the respondents included in the analyses with the general population on key sociodemographic characteristics. [file 1479-5868-10-28-S2.docx]

**Additional file 2**

Comparison of study population (N=1628) with the general population

| **Domain** | **Variable** | **Level** | **Study sample**  **(%)** | **General population (%)** | **Comparison population** |
| --- | --- | --- | --- | --- | --- |
| Demo- | Sex | Male | 46.5 | 49 | Local: Office |
| graphic |  | Female | 53.5 | 51 | for National |
|  | Age (years) | 18-29 | 14.7 | 26 | Statistics 2010^a^ |
|  |  | 30-49 | 22.1 | 35 |  |
|  |  | 50-64 | 33.6 | 22 |  |
|  |  | 65+ | 29.6 | 17 |  |
|  | Ethnicity | White | 96.4 | 94 | Local: Census |
|  |  | Non-White | 3.6 | 6 | 2001^b^ |
|  | Any child | No | 82.6 | 60 |  |
|  | under 16 | Yes | 17.4 | 40 |  |
| Socio- | Highest | Degree | 41.5 | 26 |  |
| economic | educational | A-level | 16.1 | 11 |  |
|  | qualification | GCSE | 18.5 | 16 |  |
|  |  | None or other | 23.8 | 46 |  |
|  | Tenure | Home owner | 84.4 | 70 |  |
|  |  | Renting | 15.6 | 31 |  |
|  | Employment | Employed | 54.8 | 64 |  |
|  | status | Unemployed | 1.8 | 3 |  |
|  |  | Student | 3.0 | 6 |  |
|  |  | Other economically inactive | 40.4 | 27 |  |
| Health | Weight status | Normal/underweight | 50.3 | 39 | National: Health |
|  |  | Overweight | 36.1 | 38 | survey for |
|  |  | Obese | 13.6 | 23 | England 2009^c^ |
| Travel | Cars per adult | No cars | 10.3 | 20 | Local: Census |
|  | in household | <1 car per adult | 38.8 | 35 | 2001^b^ |
|  |  | ≥1 cars per adult | 50.9 | 44 |  |

^a^ ONS mid 2010 population estimates [[23](#_ENREF_23)], percentages calculated by authors. Included in the estimates are all adult residents (aged ≥16 years) living in the three local authorities from which the study sample was drawn giving equal weighting to each local authority.

^b^ Census 2001 5% sample in Small Area Microdata [[24](#_ENREF_24)], percentages calculated by authors. Included in the estimates are all adult residents (aged >20 years) living in private households in the three local authorities from which the study sample was drawn from, giving equal weighting to each local authority. To ensure comparability, the study sample was restricted those ages 20 or more (97% of sample) when making comparisons with the census data.

^c^ Health Survey for England 2009, adult sample [[25](#_ENREF_25)]
