# Supplementary material for: Change in active travel and changes in recreational and total physical activity in adults: longitudinal findings from the iConnect study
Source: Int J Behav Nutr Phys Act. 2013 Feb 27;10:28. doi: 10.1186/1479-5868-10-28 (PMC3598920; doi:10.1186/1479-5868-10-28)
Supplement: Additional file 3 — Association between subcategories of change in active travel and change in (a) recreational and (b) total physical activity. The findings from supplementary analyses of the association between subcategories of change in active travel and (a) recreational and (b) total physical activity. [file 1479-5868-10-28-S3.docx]

**Additional file 3**

**Association between subcategories of change in active travel and change in (a) recreational and (b) total physical activity**

|  | N (%) | Recreational PA | | Total PA | |
| --- | --- | --- | --- | --- | --- |
| Change in Active Travel |  | Mean Change  (95% CI) | Regression Coefficient^a^  (95 % CI) | Mean Change  (95% CI) | Regression Coefficient^a^  (95% CI) |
| Maintained zero | 382 (23.5) | -34.2 (-63.0, -5.4) | 0 | -34.2 (-63.0, -5.4) | 0 |
| Maintained some | 152 (9.3) | 4.8 (-52.7, 62.3) | 40.7 (-46.2, 127.5) | 3.4 (-54.1, 61.0) | 42.8 (-20.5, 106.1) |
| Reduced but continued some | 358 (22.0) | -52.6 (-83.6, -21.5) | -10.2 (-56.5, 36.1) | -199.7 (-234.3, -165.1) | -166.1 (-216.7, -115.4) |
| Reduced to zero | 207 (12.7) | -17.4 (-58.8, 24.1) | 19.6 (-32.8, 72.0) | -137.4 (-182.0, -92.9) | -103.6 (-160.2, -47.0) |
| Increased from some | 187 (11.5) | -28.1 (-75.3, 19.1) | -0.5 (-55.2, 54.2) | 97.8 (46.9, 148.7) | 131.5 (72.9, 190.0) |
| Increased from zero | 342 (21.0) | -17.2 (-52.1, 17.8) | 26.0 (-20.6, 73.7) | 120.1 (82.3, 1507.9) | 155.6 (104.9, 206.2) |

^a^Linear regression adjusted for age, sex, ethnicity, education, employment, BMI, household income, housing tenure, household car access and children living at home
